# Preparation of Isotopically Labelled Standards of Creatinine Via H/D Exchange and Their Application in Quantitative Analysis by LC-MS

**DOI:** 10.3390/molecules25071514

**Published:** 2020-03-26

**Authors:** Remigiusz Bąchor, Andrzej Konieczny, Zbigniew Szewczuk

**Affiliations:** 1 Faculty of Chemistry, University of Wroclaw, 50-383 Wroclaw, Poland; zbigniew.szewczuk@chem.uni.wroc.pl; 2 Department of Nephrology and Transplantation Medicine, Wroclaw Medical University, 50-556 Wroclaw, Poland; andrzej_konieczny@yahoo.com

**Keywords:** creatinine, hydrogen/deuterium exchange, isotope dilution, liquid chromatography-mass spectrometry

## Abstract

Kidneys play a crucial role in maintaining metabolic homeostasis in a body. Serum creatinine concentration is a simple test used as an indicator of renal function. One of the known ways of quantifying creatinine concentration is the liquid chromatography-mass spectrometry (LC-MS) method, using an isotopically labeled analog of creatinine as an internal standard. Unfortunately, such isotope-labeled analogs are expensive and their synthesis is complex. Here we demonstrate a facile preparation of deuterated analogues of creatinine, via the H/D exchange of hydrogens located at the α-carbon (α-C) of the N-methylated amino acid part, under basic conditions. The stability of retrieved isotopologues was analyzed under both neutral or acidic conditions, and the results revealed that the introduced deuterons do not undergo back-exchange. In addition, the coelution of deuterated and non-deuterated forms under acidic and neutral conditions was observed. The prepared isotopologues were successfully applied in the quantitative LC-MS analysis of urine samples, and the results demonstrated that the presented strategy is novel and inexpensive, and that the quantification correlates with the commonly used Jaffe test method.

## 1. Introduction

Creatinine, 2-amino-1-methyl-1,5-dihydro-4H-imidazol-4-on, is a breakdown product of creatine phosphate in muscle. The loss of a water molecule from creatine results in the formation of creatinine as a heterocyclic compound. Creatinine is transferred to the kidneys by blood plasma, whereupon it is eliminated from the body by glomerular filtration and partial tubular excretion. Creatinine is usually produced at a fairly constant rate by the body. Measuring serum creatinine is a simple test, and it is the most commonly used indicator of renal function. A rise in blood creatinine levels is observed only with marked damage to functioning nephrons. Therefore, the detection of renal hypofunction is highly desired. The serum concentrations of creatine (sCr) and creatinine are commonly used for the determination of the renal function. Urinary creatinine (uCre) analysis may be used to calculate creatinine clearance, confirm the completeness of 24-h collections, or serve as a reference quantity for other analyses, such as in the calculation of the albumin or protein/creatinine ratio. 

There are several ways of determining both the serum and urine creatinine concentrations, such as ELISA [1], colorimetry [2], Cre biosensor [3], capillary electrophoresis with electrochemical detection [4], quantitative ^1^H-NMR [5], LC-UV [6], GC-MS and LC-MS [7]

Liquid chromatography-mass spectrometry is currently the method of choice in the quantitative analysis of Cre, due to its sensitivity, low-cost and potential for analysis of complex mixtures [8,9,10]. The LC-MS-based Cre quantification methods comprise the addition of defined quantities of isotopically-labeled standard (isotopologue) to the analyzed sample, for the absolute quantification using the isotope-dilution method [11,12]. The assessment is based on the comparison between the relative intensity of signals, corresponding to the known amount of isotopically labeled standard, and the analyzed compound. The applied isotopological internal standards should exhibit a chromatographic behavior, identical to the native compounds, but be distinguishable by their mass difference [13]. Additionally, introduced isotopes cannot undergo back-exchange during LC-MS separation conditions. Although isotope-labeled creatinine standards are commercially available (Cre-d_3_ and Cre-d_5_) and widely used for analysis [8,10], due to their complicated and expensive synthesis their price is high. Therefore, the development of new methods of isotopically-labeled creatinine standards synthesis is still under investigation. Actually, there is 1800-fold difference between the average cost of creatinine and d_3_-creatinine and the method proposed by us in this article, using new deuterated standards of creatinine. It might reduce the cost of deuterated Cre by 1000-fold.

Recently, we developed a method for the deuterium labeling of peptides containing N-substituted glycine residues, via the hydrogen/deuterium exchange (HDX) of their α-carbon hydrogen atoms, occurring in D_2_O, containing 1% *N,N,N*-trimethylamine (TEA) [14]. The deuterons introduced at α-C atom do not undergo back-exchange either in an acidic or neutral aqueous solution and are suitable for an accurate peptide quantification by mass spectrometry. We have also found that such an exchange does not occur in the case of the *N*-methylalanine residue, in model peptides [15]. The method was successfully applied for the deuterium labeling of denatonium benzoate (Bitrex) via HDX reaction at the α-carbon situated between the carbonyl and quaternary ammonium group [16]. Additionally, we developed a method of preparation of deuterium-labeled cyclosporine A standards via the HDX of their α-carbon hydrogen atoms, occurring in D_2_O under basic conditions [17]. The deuterons introduced at α-C atom do not undergo back-exchange either in an acidic or neutral aqueous solution and are suitable for an accurate peptide quantification by mass spectrometry. The proposed strategy is rapid, cost-efficient and does not require special derivatization reagents or further purification. The LC-MS analysis of denatonium cation isotopologues revealed that the introduced deuterons do not undergo back-exchange under acidic conditions and that the coelution of deuterated and non-deuterated forms was observed during standard HPLC conditions. 

However, the discovered HDX has never been applied in the preparation of isotopically labeled analogues of the creatinine molecule. Within the Cre molecule, the *N*-methylated amino acid part may be found. Therefore, two α-C hydrogens may be exchanged into two deuterons using our previous strategy. The aim of this work was to investigate the possibility of HDX of α-C hydrogens within the Cre molecule, under basic conditions, at room temperature, and to test the applicability of the prepared deuterated standard in the quantitative analysis of creatinine, using LC-MS. According to our investigations, the costs of the preparation of the deuterated Cre would be reduced by at least 100 times, because heavy water is the only deuteration reagent used in this synthesis. 

## 2. Results and Discussion

**Figure 1:**
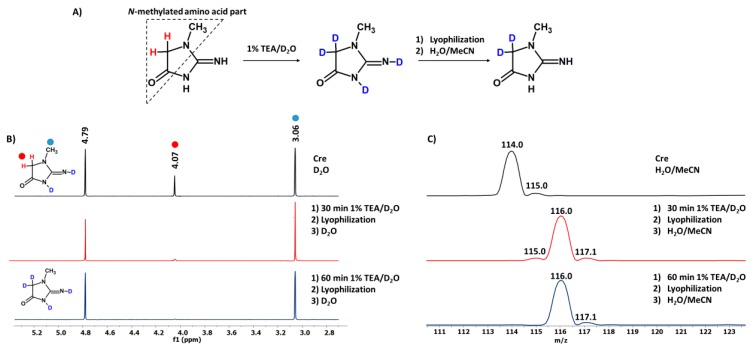
(**A**) Schematic presentation of the HDX reaction of α-C hydrogens within the *N*-methylated amino acid part in a creatinine molecule; (**B**) ^1^H-NMR analysis of Cre (upper panel), and Cre after incubation in 1% TEA/D_2_O for 30 (middle panel) and 60 min (lower panel); (**C**) ESI-MS spectra in positive ion mode of Cre dissolved in an acetonitrile-water mixture (upper panel) and after incubation in 1% TEA/D_2_O for 30 (middle panel) and 60 min (lower panel), lyophilization and sample redissolving in an acetonitrile-water mixture.

The main goal of this work was to synthesize the deuterated standards of creatinine, at α-C of the *N*-methylated amino acid part, under basic conditions (Figure 1A), to analyze the effect of deuteration on the retention times of isotopologues and to determine the applicability of the obtained standards in the quantitative analysis of Cre by LC-MS. 

According to our previous study [14,17], we tested the influence of the 1% TEA/D_2_O solution on the HDX at the α-C of the other *N*-methylated amino acid part as milder reaction conditions. In our experiment, Cre samples were incubated from 30 min to 6 hours at room temperature in the D_2_O solution containing 1% of TEA. The progress of HDX was monitored by ESI-MS and ^1^H-NMR (Figure 1B,C).

The obtained ^1^H-NMR spectra suggest that, after 60 min of HDX reaction, the signal corresponding to the α-C hydrogens at 4.07 ppm disappeared completely. Additionally, the obtained NMR spectra revealed that signals corresponding to other hydrogens of the Cre molecule persisted unchanged (3.06 ppm). After 30 min of the HDX reaction, some unexchanged fractions remained because of the presence of a low intensive signal at 115.0 *m/z* (Figure 1C, middle panel). After 60 min of incubation in 1% TEA/D_2_O solution, the isotopic distribution clearly confirms the complete HDX, due to the presence of two signals at 116.0 and 117.1 *m/z* (Figure 1C, lower panel). This suggests the possibility of introduction of two deuterium atoms even after 60 min of the reaction.

The achieved results from the NMR and ESI-MS analysis indicate a high selectivity of the analyzed process and demonstrate that the addition of TEA catalyzed the proposed reaction, at the α-C hydrogens in the Cre molecule, very rapidly. The base-catalyzed H/D exchange of the α-C hydrogens in simple aldehydes, ketones, thioesters, esters and amides [18,19,20] proceeds by a stepwise mechanism through an enolate intermediate, when the enolate is sufficiently stable to exist for the time of a bond vibration (10^–13^ s) [21]. Therefore, it may be assumed that in the case of the creatinine molecule, where the enolate at the α-C may also be formed and stabilized, the base-catalyzed H/D exchange reactions proceed via the same mechanism.

**Figure 2:**
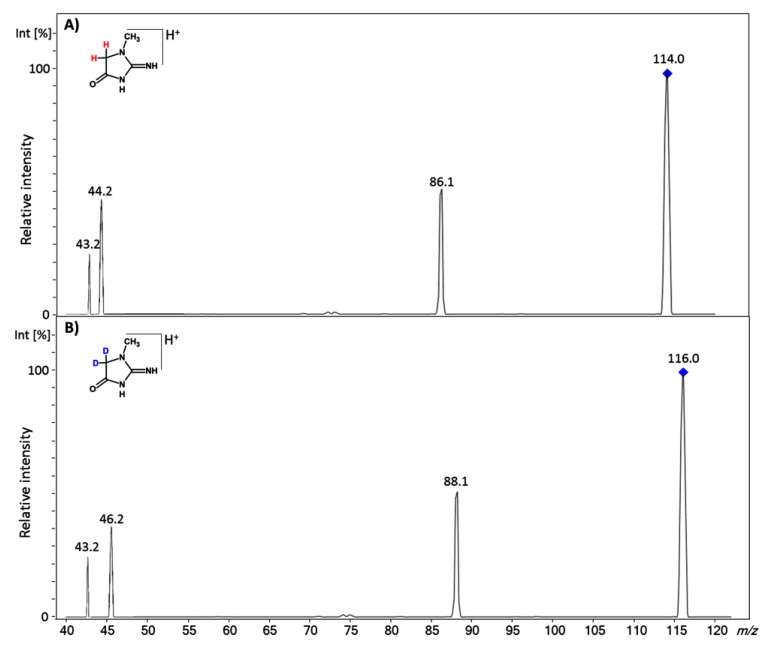
(**A**) ESI-MS/MS spectra of protonated creatinine and (**B**) the obtained deuterated creatinine isotopologue. (**A**) parent ion 114.0, collision energy 45 eV; (**B**) parent ion 116.0, collision energy 45 eV.

In our previous work [14], we observed that during the MS/MS analysis of peptide containing an *N*-methylated glycine residue, the α-C protons may undergo unexpected hydrogen scrambling. Therefore, the MS/MS experiment of the obtained deuterated creatinine isotopologue was performed, and the results are presented in Figure 2. The ESI-MS/MS analysis revealed that the introduced deuterons remained in two fragment ions at *m/z* 88.1 and 46.2 (Figure 2B), which give the pair of reference ions as shifted by 2 Da, compared to the fragmentation of the non-deuterated Cre molecule (Figure 2A). Such fragment ions may be useful for a quantitative MRM analysis, which may facilitate the analysis. Nowadays, commonly used and commercially available deuterated standards of creatinine (Cre-d_3_) give a mass shift of 3 Da and an intensive signal at *m/z* 47.2 during fragmentation, which is commonly used for MRM quantitation. However, in the case of such a small molecule like creatinine, the mass shift of 2 Da is sufficient because the intensity of the M+2 isotopic peek is negligible for compounds containing four carbon atoms only, and therefore the intensity of the monoisotopic peek from the heavy standard (d_2_) will not be affected by the intensity of the M+2 isotopic peek of the analyzed creatinine.

The acidity of the α-C hydrogens of *N*-methylated glycine analogues has been discussed before by Rios et al. [22,23] However, to the best of our knowledge, the hydrogen-deuterium exchange at the creatinine molecule, under the proposed condition, has not yet been described. The application of TEA as a volatile amine in the investigated reaction has a great advantage because it can be easily removed by lyophilization.

The introduction of two deuterons into the Cre molecule shifts the molecular mass by 2 Daltons, which may facilitate the analysis of the isotopic distribution during the quantitative analysis by LC-MS. However, to apply the obtained deuterated isotopologues of creatinine in the quantitative analysis of this immunosuppressant using LC-MS, the isotopological standards should exhibit a chromatographic behavior identical to the native compound but be distinguishable by their mass difference [13]. Of course, the introduced deuterons should also be stable under LC-MS conditions and do not undergo back-exchange. 

**Figure 3:**
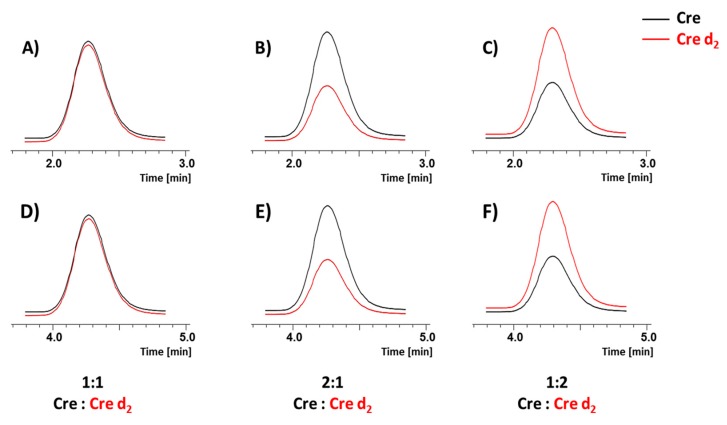
Extracted ion chromatograms of non-deuterated (**Cre**, black line) and deuterated (**Cre d_2_**, red line) creatinine samples obtained on the (**A**–**C**) C18 and (**D**–**F**) HILIC Luna column, mixed in different ratios: (**A**, **D**) 1:1; (**B**, **E**) 2:1 and (**C**, **F**) 1:2. The signal intensities correspond to the amount of isotopologues present in the analyzed samples.

Therefore, we investigated the effect of the introduction of three deuterons at the α-C in the Cre molecule on the chromatographic separation of creatinine isotopologues by LC-MS. The analysis was performed on the C18 and HILIC Luna column, and the obtained results are presented in the Figure 3. 

The extracted ion chromatograms revealed practically the same retention of deuterated and non-deuterated Cre molecules for samples containing different amounts of deuterated Cre standards (Figure 3A–C). Additionally, the intensity ratio of signals corresponding to the deuterated and non-deuterated analogues, obtained for the analyzed mixtures, confirmed the applicability of the proposed standards in the quantitative analysis of Cre. Due to the high polarity of the creatinine molecule (log P = −1.02), its retention on the hydrophobic C18 column is very low even under applied conditions. Therefore, we decided to apply the HILIC Luna column to increase the retention of the analyzed compound. The results clearly indicate that the application of the HILIC Luna column allowed the higher retention of the Cre molecule and additionally the coelution of isotopologues. (Figure 3D–F). Therefore, for the urine samples analysis, the HILIC column was used.

**Figure 4:**
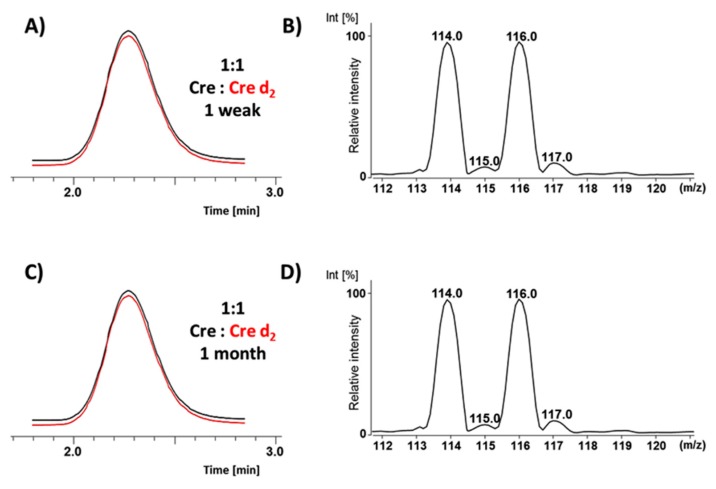
The (**A**, **C**) LC-MS and (**B**, **D**) ESI-MS analysis of the stability of the obtained deuterated Cre standards after (**A**, **B**) one week and (**C**, **D**) one month of storage in the mixture of H_2_O/MeCN/HCOOH (1:1:0.1 *v/v/v*). Extracted ion chromatograms of non-deuterated (Cre, black line) and deuterated (Cre d_2_, red line) creatinine samples mixed in a 1:1 ratio.

The stability of the synthesized deuterated Cre analogues during storage was analyzed. The samples with an equal amount of Cre and prepared Cre d_2_ isotopologues (0.1 mg) were incubated in 1 mL of H_2_O/MeCN/HCOOH (1:1:0.1 *v/v/v*) mixture, from 1 week to 1 month, under room temperature, and then analyzed by LC-MS method. The obtained spectra did not reveal any changes either in the isotopic pattern (MS spectra, Figure 4B,D) or in the signal intensities (LC-MS, Figure 4A,C), even after 1 month of incubation. The experiment showed that the deuterated Cre standards are stable at neutral and acidic pH during storage, under room temperature. 

**Figure 5:**
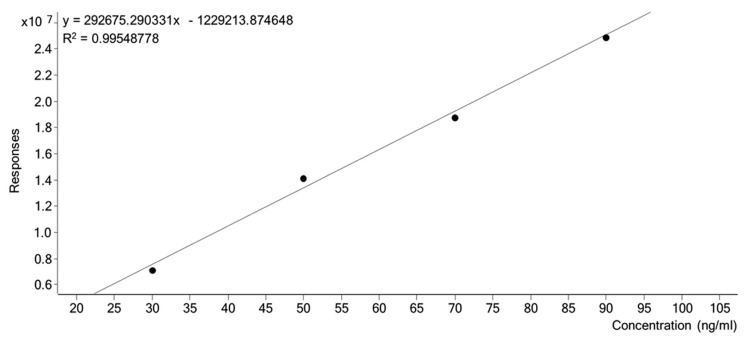
The obtained calibration curve of Cre by the LC-MS analysis of the prepared standard solutions.

To confirm the applicability of the obtained deuterated analogues of creatinine in the quantitative LC-MS analysis, the urine samples from three different patients were investigated. Fixed concentrations of Cre corresponding to 30, 50, 90 and 100 mg/dL were prepared by sequential dilutions of the stock solutions. The calibration curve (Figure 5) was constructed by plotting the peak area ratio of each compound to I.S. (y) versus the concentration of the compounds (x, mg/dL). The measurements were repeated 5 times. The results after LC-MS quantitation were compared with the amount of creatinine, determined using Jaffe test, and are presented in Table 1.

The concentration of creatinine in three different urine samples determined by the LC-MS using the obtained deuterated isotopologues is very close to the concentration of this metabolite determined using the Jaffe test. Such results clearly indicate the applicability of the proposed deuterated creatinine isotopologues as internal standards in the LC-MS quantitative analysis by isotopic dilution. Additionally, the comparison between the obtained creatinine-d_2_ and commercially available creatinine-d_3_ isotopologues in the LC-MS quantitative analysis of this metabolite was performed. The results clearly demonstrate the coelution of isotopologues and that the quantitation using Cre-d_3_ and Cre-d_2_ was at the same level in the case of the analyzed samples. 

## 3. Materials and Methods

### 3.1. Chemicals

Creatinine (**≥**98%), d_3_-creatinine, deuterium oxide (D_2_O, 99.9% purity), *N,N,N*-triethylamine (TEA), acetonitrile, formic acid and ammonium formate (LC-MS grade) were purchased from Sigma-Aldrich (Saint Louis, MO, USA), Creatinine 3L18 kit (Abbot Laboratories, Abbot Part, IL, USA).

### 3.2. Mass Spectrometry

LCMS-8050 Shimadzu apparatus (Shimadzu Corporation, Kyoto, Japan), with a UHPLC Nexera X2 system and standard ESI source. The instrument was operated in the positive-ion mode. Analyte solutions (0.1 μL) were introduced at a flow rate of 0.2 μL/min in a H_2_O/MeCN mixture (50:50, *v:v*). The instrument parameters were as follows: scan range: 70–300 *m*/*z*; drying gas: nitrogen; flow rate: 1.5 l/min; temperature: 300 °C; potential between the spray needle and the orifice: 4.2 kV.

### 3.3. Isotopic Exchange 

HDX was performed by dissolving 2.0 mg of the creatinine in 297 μL of D_2_O at room temperature. The HDX was initiated by addition of 3 μL of TEA (pD = 12.3). The sample was mixed and incubated at room temperature and lyophilized. The sample was redissolved in 200 µL of a water/MeCN (1:1, *v/v*) mixture, incubated for 30 min and subjected to an ESI-MS analysis. 

In order to estimate the pD of the analyzed alkaline solutions, the pH was measured using a MP230 pH meter (Mettler-Toledo, Greifensee, Switzerland). The pD was calculated according to the equation: pD = pH + 0.4 [24]. The obtained pD value was 12.4 for 1% TEA/D_2_O mixture at room temperature. 

### 3.4. H-NMR Analysis

NMR spectra were recorded on a high-field Bruker Avance 500 MHz NMR spectrometer (Bruker Daltonics, Bremen, Germany). A ^1^H-NMR analysis was performed for each Cre sample after HDX. Samples were dissolved in D_2_O. All NMR data are presented in the supplementary data (Figures S1–S4). 

### 3.5. Liquid Chromatography-Mass Spectrometry (LC-MS) Analysis

LC-MS/MRM experiments were performed on a LCMS-8050 Shimadzu apparatus (Shimadzu Corporation, Kyoto, Japan), with a UHPLC Nexera X2 system, equipped with an Aeris Peptide XB-C18 column (50 mm × 2.1 mm) with a 3.6 μm bead diameter and HILIC Luna (3.0 μm, 50 mm × 2.1 mm, PHENOMENEX) equilibrated at 24 °C. The LC system was operated with the following mobile phases: for the Aeris Peptide XB-C18 column (A: 0.1% formic acid in H_2_O and solvent B: 0.1% formic acid in MeCN), the gradient conditions (B %) were from 0.5% to 2% B within 10 min; for the HILIC Luna column (A: 5 mM HCOONH_4_/H_2_O and B: MeCN + 5% 100 mM HCOONH_4_/H_2_O), the gradient conditions (B %) were from 92% to 82% B within 10 min. The flow rate was 0.1 mL/min and the injection volume 0.1 μL. For MRM data analysis, LabSolutions software, version 3.0 was used. All chromatograms are presented in supplementary data (Figures S5–S10).

### 3.6. Urine Samples Preaparation

Urine samples were collected anonymously from healthy voluntary donors (mean donors age was 33 years). Middle beam urine was collected in a standard urine collection beaker. Frozen urine samples were thawed to room temperature and mixed to suspend any settled to precipitate. A 10 μL formic acid was added to 1 mL aliquot of human urine sample, stirred and centrifuged (MPW-352R Centrifuge, MPW MED. INSTRUMENTS, Poland) at 10,000 rpm for 10 min. The mixture was filtered through a 0.22 μm membrane and a 10 μL urine aliquot was transferred to a vial and brought to a total volume of 1 mL with water after being spiked with 10 μL of creatinine-d_2_ internal standard solution (1 μg/mL). A 5 μL aliquot was injected on-column for LC-MS-MS [25].

### 3.7. Urinary Creatinine Concentration Determination 

Urinary creatinine concentration was assessed with the use of commercially available kit Creatinine 3L18 (Abbot Laboratories, Abbot Part, IL, USA), based on improved Jaffe reaction [26]. The assay was performed on freshly voided morning urine sample. At the alkaline pH, creatinine in the sample reacts with picrate to form a creatinine-picrate complex. The rate of increase in absorbance at 500 nm due to the formation of this complex is directly proportional to the concentration of the creatinine in the sample.

The experimental procedures were conducted in accordance with the ethical standards of the Helsinki Declaration and were approved by the Local Bioethical Commission No. KP/No. 2/year 2015, regarding the project "Searching for new diagnostic methods in pre-eclampsia".

All patients were informed in detail about the purpose of research, process and voluntary nature of participation before giving their consent to participate in the study and reminded again at the time of data collection. 

## 4. Conclusions

We presented a new method of synthesis of deuterated creatinine analogues, using organic base and D_2_O. The study allowed us to obtain the Cre analogue, containing two deuterons, which were introduced after 60 min incubation in a 1% solution of TEA in D_2_O at room temperature. The introduced deuterons did not undergo back-exchange under acidic and neutral conditions, which makes the obtained isotopologue a good internal standard for quantitative analysis by ESI-MS using the isotope dilution method. Additionally, it was found that the isotopologues were characterized by practically the same retention time during chromatographic separation, which may be especially useful during the LC-MS analysis of Cre-containing samples. The results demonstrate that the presented strategy is a new, inexpensive and simple solution for Cre quantification, and the quantification correlates with the Jaffe test method.

## Figures and Tables

**Table 1:** Amount of creatinine in the urine samples from three different patients determined by the LC-MS method and Jaffe test.

URINE	Cre Concentration (mg/dL)	
SAMPLE	LC-MS	Jaffe test
**PATIENT 1**	27.6	29.5
**PATIENT 2**	51.7	49.4
**PATIENT 3**	63.8	61.1

## Supplementary Materials

The following are available online at, Figure S1. ^1^H-NMR spectrum of creatinine dissolved in D_2_O; Figure S2. ^13^C-NMR spectrum of creatinine dissolved D_2_O; Figure S3. ^1^H-NMR spectrum of creatinine after incubation in 1% TEA/D_2_O for 60 minutes. D_2_O was used as a solvent; Figure S4. ^13^C-NMR spectrum of creatinine after incubation in 1% TEA/D_2_O for 60 minutes in D_2_O; Figure S5. Extracted ion chromatograms of non-deuterated (**Cre**, black line) and deuterated (**Cre d_2_**, red line) creatinine samples obtained on HILIC Luna column mixed in 1:1 ratio; Figure S6. Extracted ion chromatograms of non-deuterated (**Cre**, black line) and deuterated (**Cre d_2_**, red line) creatinine samples obtained on HILIC Luna column mixed in 2:1 ratio; Figure S7. Extracted ion chromatograms of non-deuterated (**Cre**, black line) and deuterated (**Cre d_2_**, red line) creatinine samples obtained on HILIC Luna column mixed in 1:2 ratio; Figure S8. Extracted ion chromatograms of non-deuterated (**Cre**, black line) and deuterated (**Cre d_2_**, red line) creatinine samples obtained on Aeris Peptide XB-C18 column column mixed in 1:1 ratio; Figure S9. Extracted ion chromatograms of non-deuterated (**Cre**, black line) and deuterated (**Cre d_2_**, red line) creatinine samples obtained on Aeris Peptide XB-C18 column column mixed in 2:1 ratio; Figure S10. Extracted ion chromatograms of non-deuterated (**Cre**, black line) and deuterated (**Cre d_2_**, red line) creatinine samples obtained on Aeris Peptide XB-C18 column column mixed in 1:2 ratio.

## Founding

This work was supported by the grant No. UMO-2016/23/B/ST4/01036 from the National Science Centre, Poland.
