# Supplementary material for: Preparation of Isotopically Labelled Standards of Creatinine Via H/D Exchange and Their Application in Quantitative Analysis by LC-MS
Source: Molecules. 2020 Mar 26;25(7):1514. doi: 10.3390/molecules25071514 (PMC7180833; doi:10.3390/molecules25071514)
Supplement: Supplementary file 1 [file molecules-25-01514-s001.pdf]

Preparation of isotopically labelled standards of creatinine via H/D exchange and their application in quantitative analysis by LC-MS

Remigiusz Bąchor[1]*, Andrzej Konieczny[2] and Zbigniew Szewczuk[1]

[1]Faculty of Chemistry, University of Wroclaw, 50-383 Wroclaw, Poland

[2] Wroclaw Medical University, Department of Nephrology and Transplantation Medicine, 50-556 Wroclaw, Poland;

* Correspondence: Corresponding: Remigiusz Bąchor, Faculty of Chemistry, University of Wroclaw, F. Joliot-Curie 14, 50-383 Wroclaw, Poland, Fax: +48 71 328 2348, Tel.: +48 71 375 7218, E mail: remigiusz.bachor@chem.uni.wroc.pl

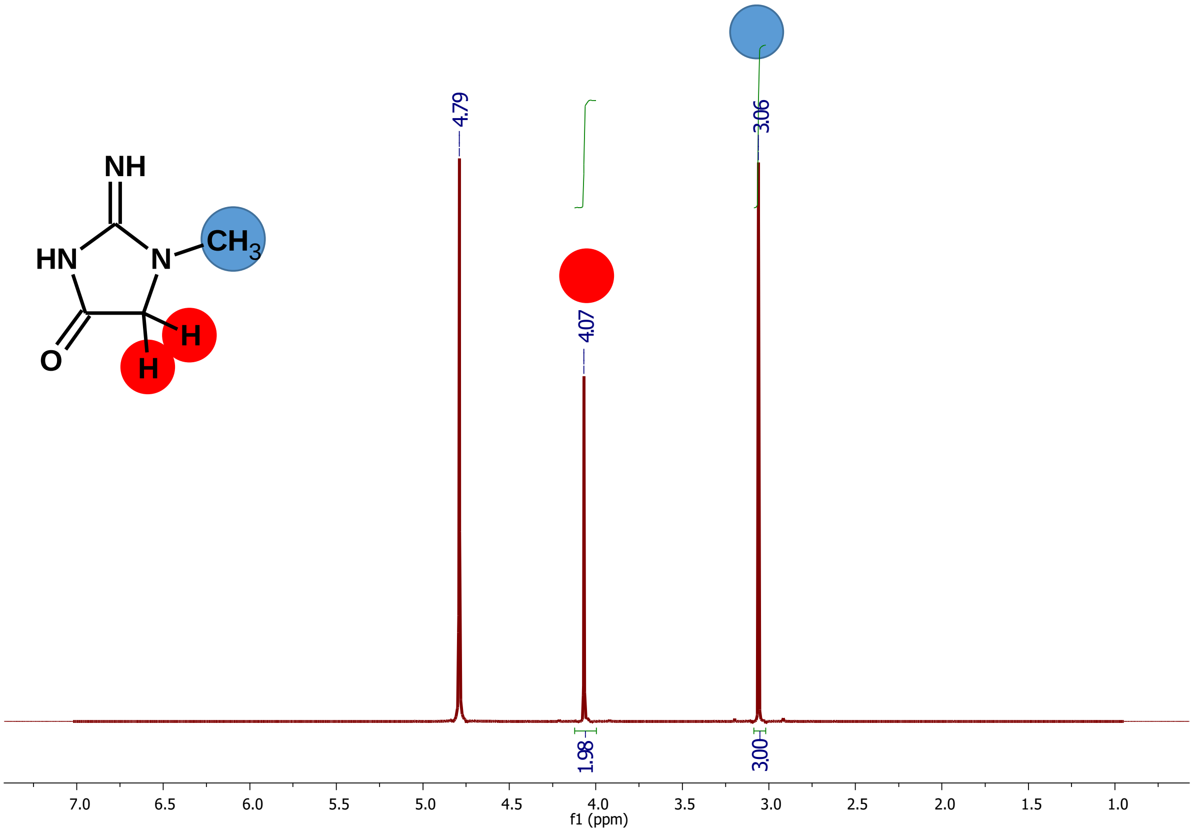

Figure 1S. ^{1}H-NMR spectrum of creatinine dissolved in D_2O.

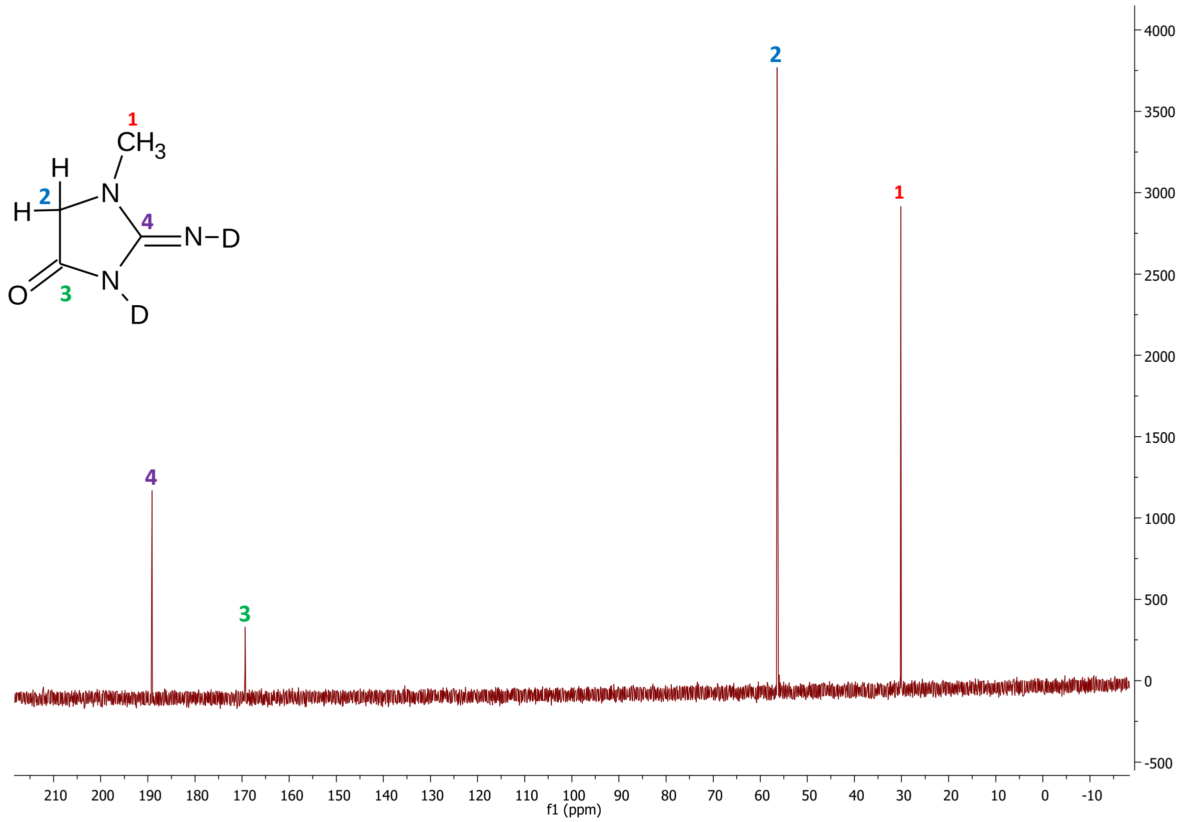

Figure 2S. ^{13}C-NMR spectrum of creatinine dissolved D_2O.

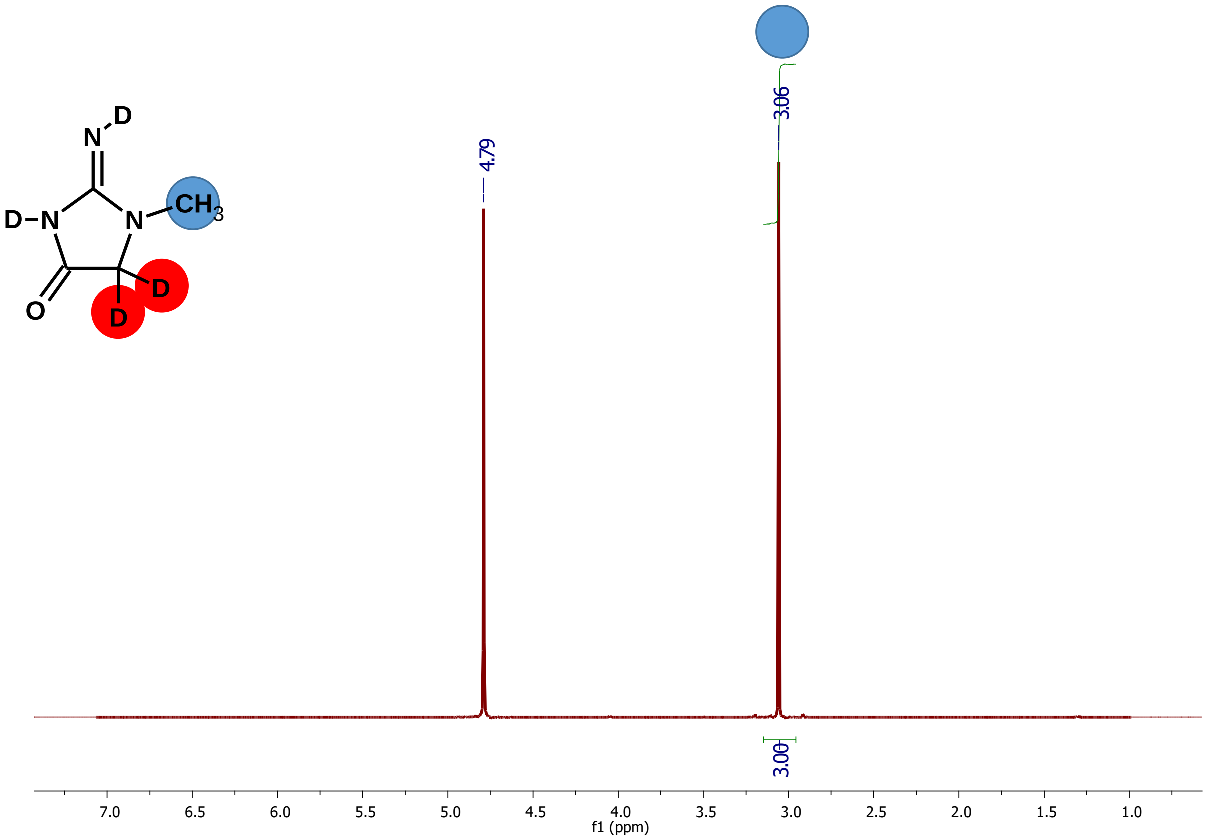

Figure 3S. ^{1}H-NMR spectrum of creatinine after incubation in 1% TEA/D_2O for 60 minutes. D_2O was used as a solvent.

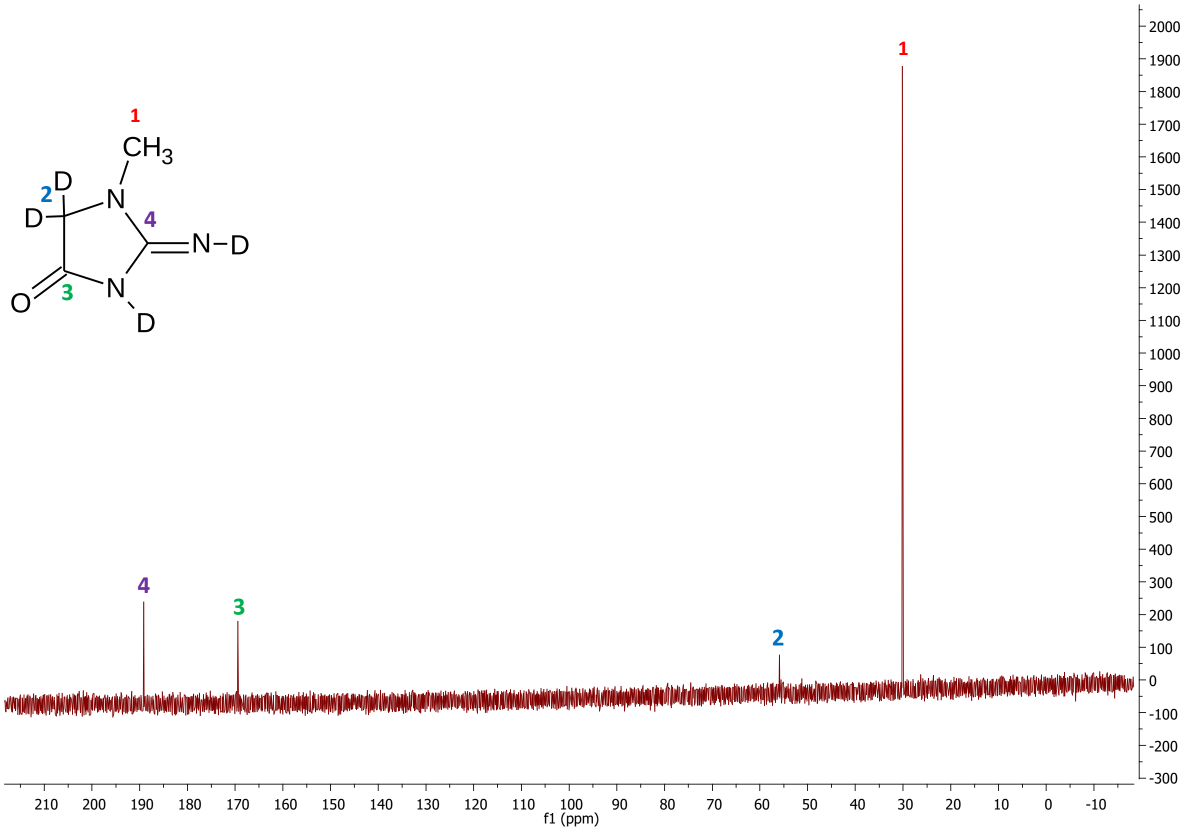

Figure 4S. ^{13}C-NMR spectrum of creatinine after incubation in 1% TEA/D_2O for 60 minutes in D_2O.

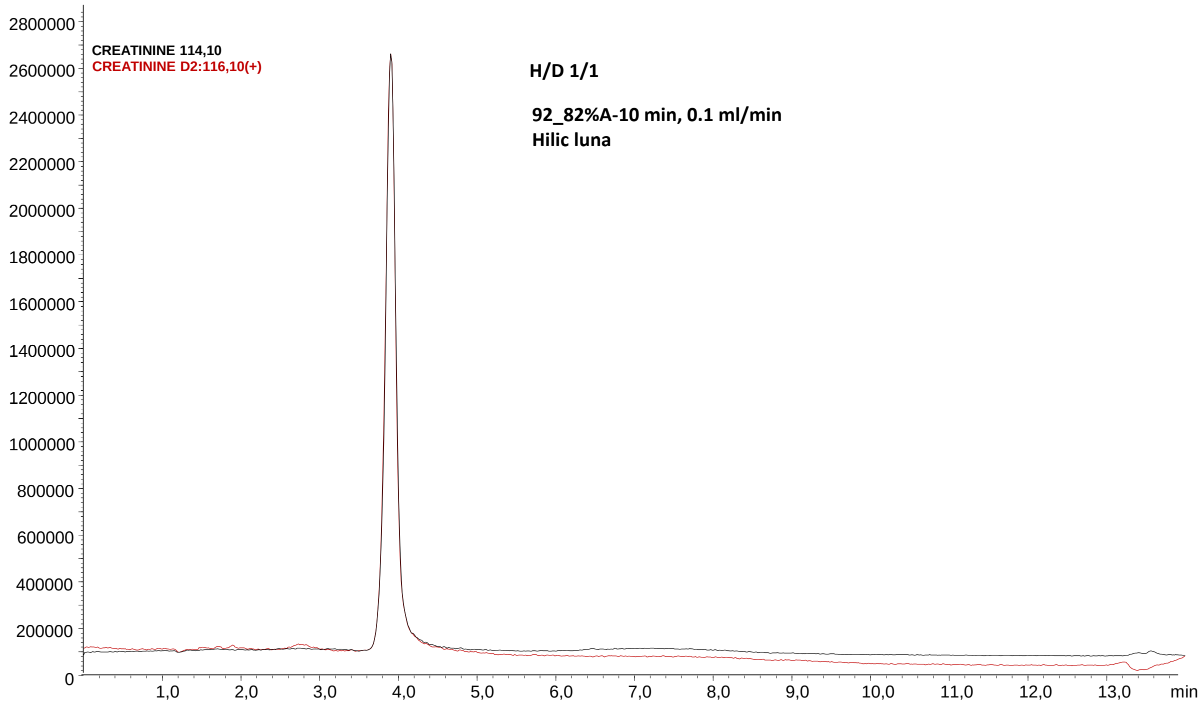

Figure 5S. Extracted ion chromatograms of non-deuterated (**Cre**, black line) and deuterated (**Cre d$_2$**, red line) creatinine samples obtained on HILIC Luna column mixed in 1:1 ratio.

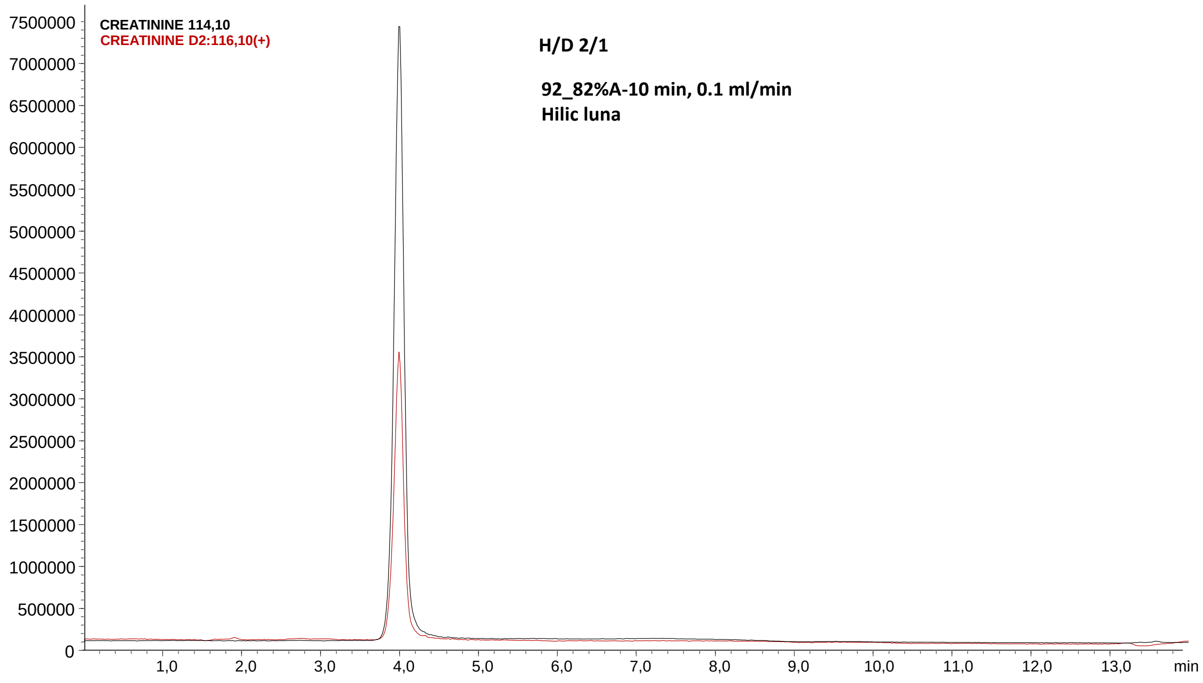

Figure 6S. Extracted ion chromatograms of non-deuterated (**Cre**, black line) and deuterated (**Cre d$_2$**, red line) creatinine samples obtained on HILIC Luna column mixed in 2:1 ratio.

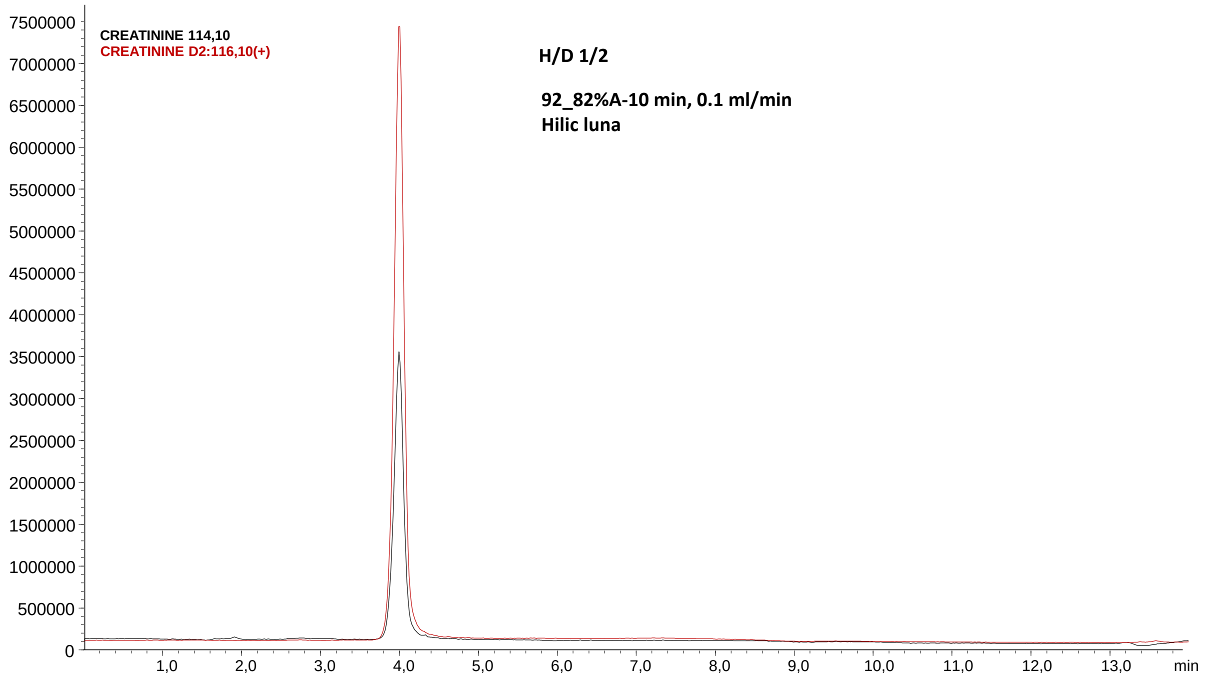

Figure 7S. Extracted ion chromatograms of non-deuterated (**Cre**, black line) and deuterated (**Cre d$_2$**, red line) creatinine samples obtained on HILIC Luna column mixed in 1:2 ratio.

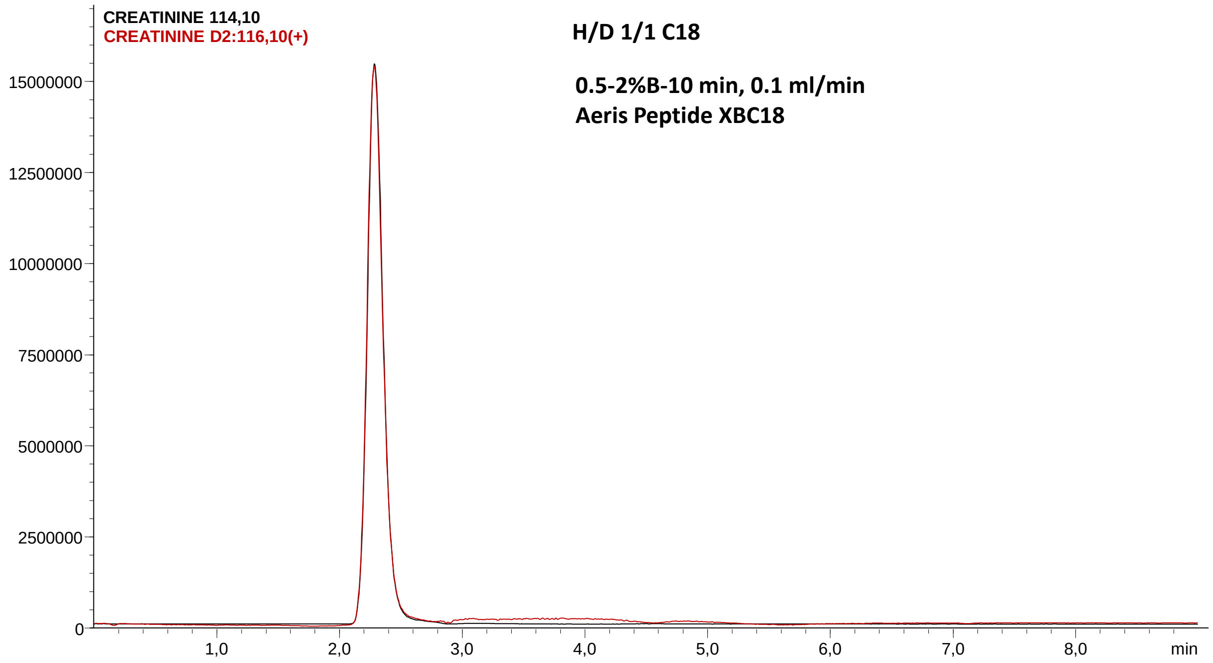

Figure 8S. Extracted ion chromatograms of non-deuterated (**Cre**, black line) and deuterated (**Cre d$_2$**, red line) creatinine samples obtained on Aeris Peptide XB-C18 column column mixed in 1:1 ratio.

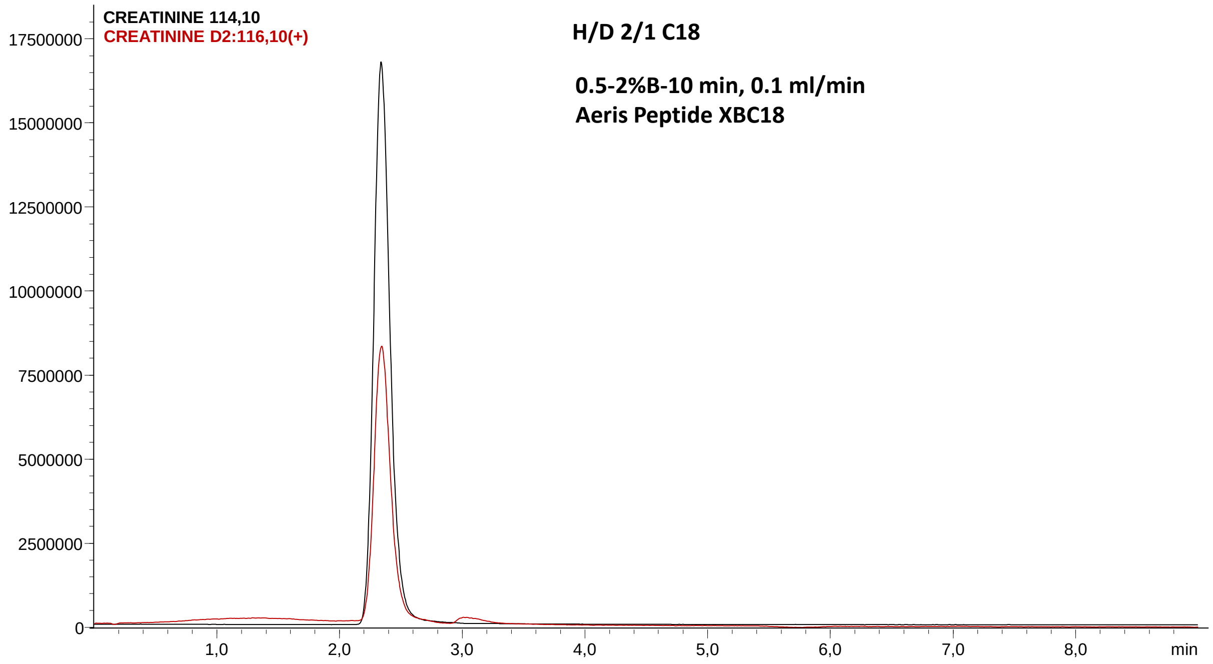

Figure 9S. Extracted ion chromatograms of non-deuterated (**Cre**, black line) and deuterated (**Cre d2**, red line) creatinine samples obtained on Aeris Peptide XB-C18 column column mixed in 2:1 ratio.

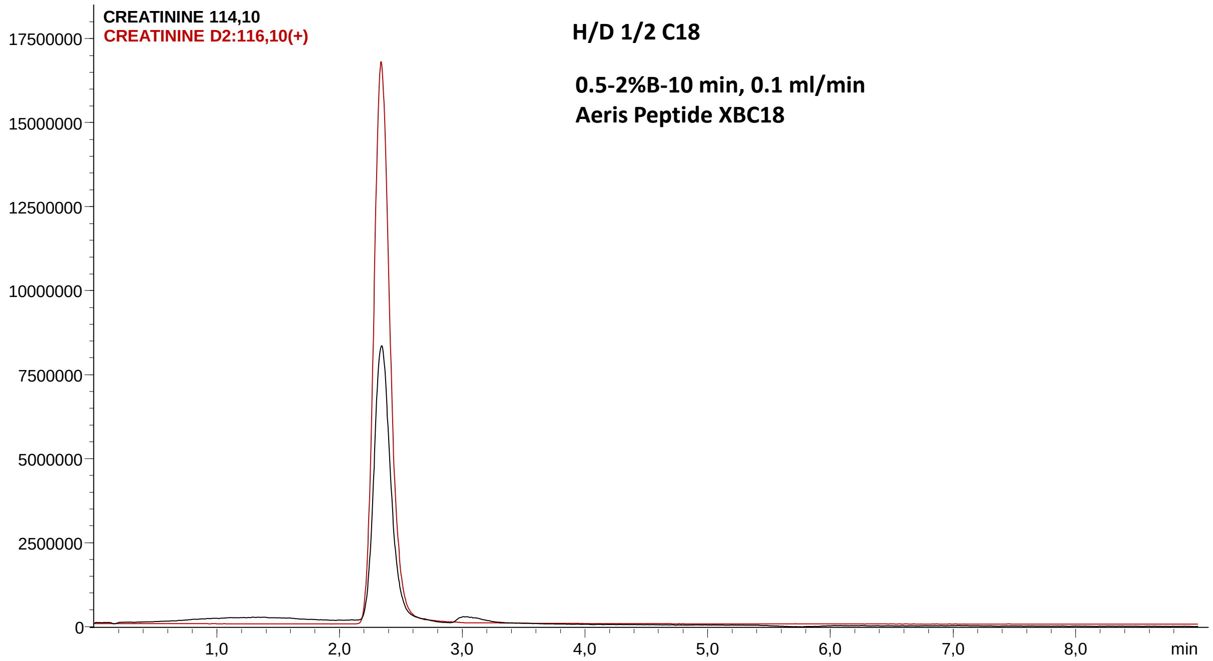

Figure 10S. Extracted ion chromatograms of non-deuterated (**Cre**, black line) and deuterated (**Cre d2**, red line) creatinine samples obtained on Aeris Peptide XB-C18 column column mixed in 1:2 ratio.
